# Pharmacoepidemiology for nephrologists: do proton pump inhibitors cause chronic kidney disease?

**DOI:** 10.1093/ndt/gfw349

**Published:** 2017-02-11

**Authors:** Laurie A. Tomlinson, Damian G. Fogarty, Ian Douglas, Dorothea Nitsch

**Affiliations:** 1 Faculty of Epidemiology and Population Health, London School of Hygiene and Tropical Medicine, Keppel Street, London WC1E 7HT, UK; 2 Department of Nephrology, Belfast Health and Social Care Trust, Belfast, Northern Ireland, UK

**Keywords:** bias, chronic kidney disease, observational studies, pharmacoepidemiology, proton pump inhibitors

## Abstract

Pharmacoepidemiology studies are increasingly used for research into safe prescribing in chronic kidney disease (CKD). Typically, patients prescribed a drug are compared with patients who are not on the drug and outcomes are compared to draw conclusions about the drug effects. This review article aims to provide the reader with a framework to critically appraise such research. A key concern in pharmacoepidemiology studies is confounding, in that patients who have worse health status are prescribed more drugs or different agents and their worse outcomes are attributed to the drugs not the health status. It may be challenging to adjust for this using statistical methods unless a comparison group with a similar health status but who are prescribed a different (comparison) drug(s) is identified. Another challenge in pharmacoepidemiology is outcome misclassification, as people who are more ill engage more often with the health service, leading to earlier diagnosis in people who are frequent attenders. Finally, using replication cohorts with the same methodology in the same type of health system does not ensure that findings are more robust. We use two recent papers that investigated the association of proton pump inhibitor drugs with CKD as a device to review the main pitfalls of pharmacoepidemiology studies and how to attempt to mitigate against potential biases that can occur.

## INTRODUCTION

**FIGURE 1:**
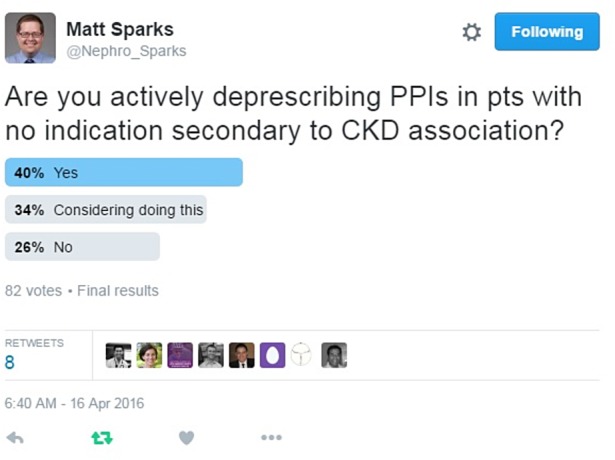
The relationship between PPIs and CKD has caused much discussion on social media [6] (colour image available online).

Two recent papers reporting an association between proton pump inhibitors (PPIs) and incident chronic kidney disease (CKD) [1, 2] have led to much discussion among nephrologists. Although the limitations of these studies have been mentioned by the authors of the papers and discussed in editorials [3–5], anecdotally these studies are leading to a change in clinical practice (Figure 1). These studies are well-conducted, thoughtful analyses with many strengths. The aim of this article is not to criticize them, but to highlight the caveats that need to be considered when drawing causal conclusions from associations reported in pharmacoepidemiological studies. We have not considered every nuance of the papers, but focused on areas that are important for the broader principles.

The results of an epidemiological study linking a medication with a health outcome can be explained in four ways. First, there may indeed be a causal association between PPIs and the development of CKD. The second possibility is that the results may have arisen by chance. However, the large sample sizes provided by prescribing and billing databases usually provide substantial statistical power for the planned analyses. Of greater concern is the third reason, namely, confounding as an alternative explanation for the observed association. Finally, other systematic error(s), e.g. differential follow-up between comparison groups, can result in an incorrect assessment of the association. Combined with high statistical power, these biases may lead to a final estimate with a low P-value and very narrow confidence intervals: a very precise association, but not a causal one [7]. These errors may be difficult to overcome unless the necessary data to control for such biases have been collected and accounted for in the analysis. Most data sources from routine care are patchy with regard to variables important to renal outcomes [8]. Therefore, caution is needed when reviewing renal pharmacoepidemiology studies.

## STUDY DESIGN

**FIGURE 2:**
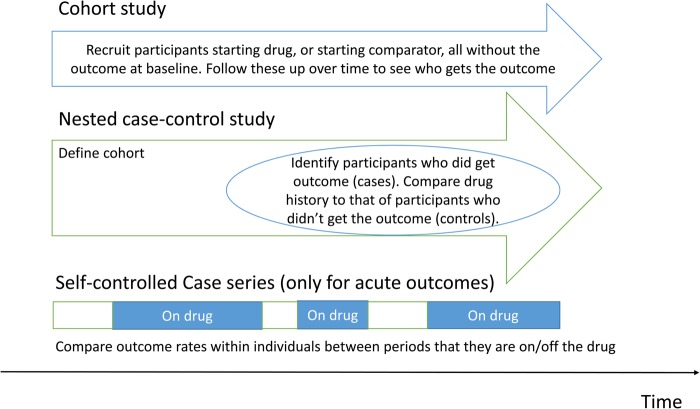
Schema of different study designs in pharmacoepidemiology (colour image available online).

It is appropriate to remind ourselves of the possible designs that a study may adopt to assess the effects of a drug exposure on a disease outcome (Figure 2). Cross-sectional studies cannot confirm a sequence of events in terms of drug exposure and outcome and are not discussed. Case–control studies are usually nested in a defined cohort to avoid selection bias. The recently published studies exploring the relationship between PPI use and risk of CKD have used cohort designs (Table 1). Because the chosen outcome was CKD, at baseline there should be no participant having CKD.

**Table 1.** Study design features of pharmacoepidemiology studies published by Lazarus *et al.* [1], and Xie *et al.* [2] that investigated the association of PPI use and CKD as well as AKI

	Lazarus *et al.*		Xie *et al.*
Study design features	ARIC study	Geisinger Health System	Department of Veterans Affairs national databases
Population studied	ARIC study participants with eGFR ≥60 mL/min/1.73 m^2^ and complete baseline data (at visit 4 of the original ARIC study)	eGFR ≥60 mL/min/1.73 m^2^ documented and available blood pressure result	eGFR >60 mL/min/1.73 m^2^ in the 90 days before receiving the first PPI prescription and at least one eGFR after the prescription. (slightly different for H2-blocker users, see below)
Year of entry into cohort	1996–99	1997–2014	2006–08
Prevalent or incident drug exposure	PPI prevalent users	PPI prevalent users, PPI new users sensitivity analysis	PPI new users
Definition of drug exposure	Direct visual inspection of pill bottles for all medications used during the preceding 2 weeks	PPI prescription within 90 days before baseline	At least one PPI prescription between October 2006 and September 2008
Comparison cohort	Nested within the study population, on H_2_ blockers or PPI non-users	Nested within the study population, on H_2_ blockers or PPI non-users	eGFR >60 mL/min/1.73 m^2^ in the 90 days before H_2_-blocker prescription and at least one eGFR after the prescription. H_2_-blocker users could not have received a PPI prior to 2006. Participants could not be defined as H_2_-blocker users if they subsequently received a PPI prescription during follow-up.
Outcome definition	Incident CKD and AKI were defined by ICD-9 coding at discharge, death (ICD-10 code) or by incident ESRD, as determined through linkage with the USRDS registry	Incident CKD was defined as the first outpatient eGFR <60 mL/min/1.73 m^2^ that was sustained at all subsequent assessments of the eGFR, or ESRD defined through USRDS linkage. AKI was defined by ICD-9 coding.	First eGFR <60 mL/min/1.73 m^2^ (encompassing both CKD and AKI), and CKD defined as two eGFRs <60 mL/min/1.73 m^2^ at least 90 days apart. AKI defined as 50% serum creatinine increase or 0.3 mg/dL change within 30 days.
Confounders adjusted for	Study centre, age, gender, race, education, health insurance, household income, eGFR/urinary ACR at baseline, smoking status, BMI, systolic blood pressure, hypertension, diabetes, cardiovascular disease, concomitant medication use including antihypertensives, anticoagulants and NSAIDs	Age, gender, race, eGFR at baseline, smoking status, BMI, systolic blood pressure, hypertension, diabetes, cardiovascular disease, concomitant medication use including antihypertensives, statins, aspirin, anticoagulants and NSAIDs	Age, gender, race, eGFR at baseline, hypertension, diabetes, chronic lung disease, peripheral artery disease, cardiovascular disease, cerebrovascular disease, dementia, hepatitis C, HIV, gastro-oesophageal reflux disease, upper GI bleeding, ulcer disease, *Helicobacter pylori* infection, Barrett oesophagus, achalasia, stricture, oesophageal adenocarcinoma. In sensitivity analyses: NSAID use, urinary ACR, serum bicarbonate, ACE-I/ARB use.

BMI, body mass index; ACR, albumin:creatinine ratio; NSAIDs, non-steroidal anti-inflammatory drugs; ACE-I, angiotensin-converting enzyme inhibitor; ARB, angiotensin receptor blocker.

### Definition of the cohort

In the paper by Lazarus *et al.* [1], the first cohort is defined by participation in the Atherosclerosis Risk in Communities (ARIC) study, although with some limitations to ensure normal baseline eGFR and data completeness. This study has all the strengths of a well-designed prospective cohort, including thorough measurement of baseline covariates such as proteinuria (often poorly measured in routine data). In contrast, the second cohort is based within the Geisinger Health System (Pennsylvania, USA), a data source that provides large power but has the issues of incomplete and biased recording typical of routinely collected health care data [8]. Participants entered when they first had measurements of both creatinine (equating to an eGFR >60 mL/min/1.73 m^2^) and systolic blood pressure available. In the paper by Xie *et al*. [2], participants (US veterans) had to have an eGFR >60 mL/min/1.73 m^2^ in the 90 days before receiving the first PPI prescription and a subsequent eGFR. This restriction is important to identify those without CKD. These definitions promote inclusion of participants who are being closely monitored, which may affect the generalizability of the study, but unless monitoring is different between the comparison groups, there should be limited bias.

### Prevalent versus new-user (incident) cohort

Inclusion of existing users of a drug to define comparison groups may create bias since participants who remain on treatment have not yet developed substantial side effects or have a different indication to remain on the drug versus short-term users. Sometimes, even protective associations can be found, as for example in studies of hormone replacement therapy and vascular outcomes in women [9]. Conversely, people who remain on long-term treatment may be more sick than those who only take treatments for short periods, and therefore are generally more likely to develop conditions like acute kidney injury (AKI) or CKD. Therefore, ‘new-user cohorts’ are preferred in pharmacoepidemiology, particularly to examine side effects, such as AKI, that may occur shortly after starting treatment with a drug [10]. The paper by Lazarus *et al.* [1] is based on prevalent PPI use but includes a new-user sensitivity analysis, while the paper by Xie *et al.* [2] is based on a new-user cohort.

### Choice of comparison group

The gold-standard way of determining drug efficacy and adverse effects is the randomized clinical trial. Fundamentally this is because, if adequately powered and with a successful allocation procedure, randomization ensures that there is a balance of both measured and unmeasured confounders between the study arms. Similarly, comparison of effects between drugs, or between treatment and none, is also commonly assessed in observational studies. It can provide robust results reproducing clinical trial outcomes if there is a high degree of randomness between choices of drugs [11]. However, as use of a type of medication becomes widespread and when drugs are clearly indicated for specific conditions, the results of these study designs can be misleading. Such ‘confounding by indication’ may explain in part many drug-related adverse outcomes seen in observational studies.

The study question was whether PPI users are more likely to develop CKD than people not using PPIs. It is probable that people who do not use PPIs are less ill than PPI users. Therefore, both of these studies (at least in part) compare outcomes between users of PPIs and H_2_-blockers and find an increased incidence of CKD among those exposed to PPIs. But, although prescribed for similar indications, to what extent are H_2_-blockers a valid comparator for PPI users? We need to consider the patterns of use of each class of drug at the time that patients entered the study and compare the measured characteristics of each group.

In the primary analysis of the ARIC cohort by Lazarus *et al.* [1], patients were included between 1996 and 1999. However, there are very few PPI users (*n* = 322) in the main analysis and the date of entry (1996–99) limits the generalizability to current clinical care. What are the factors that would have led to being prescribed each class of drug at this time, when PPIs had been available for a shorter period and were still under patent? New and expensive drugs are often channelled to sicker patients for whom more familiar established treatments may have failed.

In the paper by Xie *et al.* [2], there was a fixed window for inclusion into the cohort between 2006 and 2008. There were marked differences in the proportion of users of each class identified to have indications for prescribing (e.g. 4.6% of PPI users had codes indicating upper gastrointestinal (GI) tract bleeding compared with 1.2% of H_2_-blocker users). Participants defined as H_2_-blocker users could not have received a PPI prior to 2006, whereas the PPI users could have received an H_2_-blocker. Since the study design meant that H_2_-blocker users could not have been prescribed a PPI previously, why would a patient be prescribed an H_2_-blocker first line, many years after PPIs were introduced? Cost may have been relevant but is not commented on. In addition, participants could not be defined as H_2_-blocker users if they ever subsequently received a PPI prescription. Both of these criteria bias substantially towards H_2_-blocker users being healthier than PPI users. Use of ‘future information’ to define comparison groups is problematic, as it reduces comparability to a randomized trial and can also create complex time-related biases [12]—in this case excluding from the H_2_-blocker comparison group patients who required more aggressive antisecretory therapy and who are more likely to have multimorbidity.

In both studies it seems likely that PPIs were prescribed for those with more severe upper GI comorbidities such as ulceration and bleeding, which were also indicators of poorer health status. This may be associated with a greater risk of subsequent CKD. Since prescribing indications are not available for the ARIC participants, this difference in health status is not clear from the comparison of baseline characteristics, but the statement that H_2_-blockers were ‘prescribed for the same indication as PPIs’ is certainly questionable.

To understand whether differences at baseline between the two groups will affect the results of the study, the question now is whether the two groups are otherwise similar in every important aspect with regard to their risk of the outcome. This requires a detailed understanding of the risk factors for the outcome.

### Outcome definition

For the ARIC cohort, incident CKD and AKI were defined by International Classification of Diseases, Ninth Revision (ICD-9) coding or by incident ESRD, as determined through linkage with the United States Renal Data System (USRDS) registry. Defining incident CKD with an ICD-9 code requires a patient to be admitted to hospital to get a diagnosis. Patients who are admitted to hospital need to be able to afford testing and/or admission, and so there may be socio-economic factors that prevent a timely diagnosis of CKD. Also, if PPI users were sicker and more often admitted to hospital than users of H_2_-blockers, then they may have been more likely to receive an ICD-9 code of CKD.

For the Geisinger Health System cohort, incident CKD was defined as the first outpatient eGFR <60 mL/min/1.73 m^2^ that was sustained at all subsequent assessments of the eGFR, or ESRD defined through USRDS linkage. This outcome is specific for the development of sustained CKD.

In the paper by Xie *et al.* [2], the primary outcomes were the first eGFR <60 mL/min/1.73 m^2^ (encompassing both CKD and AKI), and CKD defined as two eGFRs <60 mL/min/1.73 m^2^ at least 90 days apart.

Use of a CKD end point based on two measurements requires patients to stay alive for the second measurement to fulfil the chronicity definition. If sicker patients die beforehand or do not attend for repeat tests, they would not be coded as having CKD, which would bias the study towards the null. On the other hand, physicians may be more likely to monitor renal function regularly in those they consider to be at higher risk.

Both papers also examined an association between PPI exposure and AKI as an additional outcome. Demonstrating an association with a second outcome may substantially strengthen an argument for causality in a pharmacoepidemiological analysis, particularly where the second outcome may be on the causal pathway as is proposed for AKI and CKD. However, in these studies the outcomes are closely correlated: in the paper by Lazarus *et al*. [1], both are defined by ICD-9 coding, while in that by Xie *et al*. [2], both CKD and AKI are defined by the change in creatinine values. For the hospital-derived coding of AKI there has been a huge change in recognition (similar to that of CKD) over the past decade [13]. Determining AKI by changes in creatinine appears slightly more robust (than clinical coding), but the problem of sicker patients being more closely monitored remains. Within each cohort, the same problems apply to both outcomes, respectively. Therefore, it is unsurprising that an association between PPI use and AKI is found in both papers.

## STATISTICAL ANALYSIS

As observational data are not derived from a randomized controlled study, we cannot be sure that at baseline PPI users are directly comparable to users of H_2_-blockers in every respect. If at baseline there are systematic differences between the two groups, and these systematic differences affect the outcome either through being a risk factor for the outcome (confounding) or through differential outcome assessment (outcome misclassification), then there is scope for bias.

### Adjusting for confounding

The Lazarus *et al.* [1] study demonstrated that age, gender, race, eGFR at baseline, BMI, hypertension (and antihypertensive drug prescription), cardiovascular disease (and prescription of aspirin and statins) and anticoagulant use were all associated with PPI use. Many of these are also risk factors for CKD. In addition, CKD is associated with socio-economic status [14], and the Geisinger cohort did not have data on health insurance. The study by Xie *et al.* [2] had more data on variables associated with drug indication at baseline, but had to use proxy variables to capture associations with CKD risk, e.g. instead of smoking status, adjustments were made for chronic lung and peripheral vascular disease, and instead of health insurance status, other co-variates had to indirectly capture deprivation. The question is whether these variables are sufficient and well measured enough to fully adjust for confounding. The authors of both papers have attempted to understand some of these issues with additional sensitivity analyses.

### Propensity score methods

Both studies include analyses using a propensity score to attempt to adjust for confounding. This analytical technique examines the effect of the treatment among patients who have the same predicted probability of receiving the drug, based on their characteristics when the treatment is chosen. In short, comparisons are made between people who are more similar with regard to factors associated with receiving the drug, and therefore confounding should be reduced. If the propensity score is developed based on the same covariates as those included in the primary analysis, there is little evidence that propensity methods yield substantially different or more accurate estimates than those seen after adequate standard multivariable adjustment [15]. In both types of analysis, elimination of confounding depends on high-quality data and inclusion of all confounders in the analysis. The main benefit of using propensity scores is to identify two comparable (matched) groups at baseline. A standard regression would give an answer in the full study population but may inadvertently extrapolate beyond the regions of the data where there is a reasonable comparison to be made. In the paper by Xie *et al.* [2] (and probably also that by Lazarus *et al.* [1]) there was a large reduction in the number of PPI users included in the analysis due to individual matching to H_2_-blocker users. The reduced numbers could suggest that H_2_-blocker users are in general not comparable to PPI users in routine care, but we do not know which kind of PPI user was left out of the analysis. Reduced comparator numbers also mean that there is substantially reduced statistical power and that included PPI users may have been unrepresentative of PPI users as a whole.

### Study era

During the time periods covered by these studies there have been extensive changes in the patterns of use of acid-suppressant drugs, with PPIs being increasingly prescribed [1], and, in addition, the definitions and recording of AKI [13] as well as CKD have changed. In 1999, the Modification of Diet in Renal Disease study was published [16] and the Kidney Disease Outcomes Quality Initiative classification was published in 2002 [17]. As familiarity with the CKD staging system has grown, the sensitivity of ICD-9 codes for detection of CKD have progressively improved [18]. Both papers used Cox regression to model their final estimates, and adjustment for the duration of follow-up within the study is intrinsic to this technique. If substantial temporal changes affecting data used in an analysis are suspected, it is common to also adjust for study era and to conduct sensitivity analyses to examine whether effect estimates are similar for different periods within the study [19]. However, it does not appear that either of these studies also adjusted for calendar time. Both in the Geisinger cohort and the Xie *et al.* [2] study, adjustment for available confounders reduced the effect size. Would further adjustment for other risk factors (not captured here), including study era, attenuate the relative risk further?

### Time-updated exposure status

In a study of drug effects, people are commonly divided for analysis into groups based on their drug exposure at baseline (when they enter the cohort). This is the method used for the primary analysis of both papers, but it can introduce error since exposure status may change over the follow-up time. However, in a secondary analysis in the paper by Lazarus *et al.* [1], exposure was also modelled as a time-varying ever-use variable in which a participant who was a non-user at baseline switched categories at the first instance of PPI use. The nature of the data meant that the authors could define when patients started taking the drugs, but not when they stopped, so all subsequent time was classified as exposed even if they had stopped taking PPIs. Although this is an important step in reducing misclassification, if people taking PPIs are sicker than the comparator groups, this analysis will not reduce confounding. Ideally this analysis should also define periods of time when people have definitely stopped taking the drugs, i.e. have not been prescribed a repeat drug dose, to assess the rate of CKD during subsequent periods of better health.

### Dose response effect

Showing that increasing doses of a drug are associated with progressively greater risk of the outcome can strengthen arguments for causality in a pharmacoepidemiological study. Both studies considered here suggest a relationship between the PPI dose and the incidence of CKD for twice-versus once-daily dosing in the study by Lazarus *et al.* [1] and for duration of exposure in the paper by Xie *et al.* [2]. However, these findings may also be due to confounding: dose frequency may reflect the severity of the indication while the duration of exposure may suggest an ongoing indication for the drug, both factors that may be associated with a greater risk of subsequent CKD.

## WHAT NEXT?

Both papers produce similar estimates for the increase in risk of incident CKD among PPI users: in the adjusted models for the main analysis the hazard ratio was 1.50 [95% confidence interval (CI) 1.14–1.96] in that by Lazarus *et al.* [1] and 1.28 (95% CI 1.23–1.34) in the paper by Xie *et al.* [2]. If truly causal, these effect sizes would be very important for public health due to the frequency of prescription of PPIs.

A link between PPIs and CKD or AKI is plausible given the probable link between PPI use and interstitial nephritis [20]. However, PPIs have a long history of being associated with other illnesses. The paper by Lazarus *et al.* [1] states that ‘observational studies have linked PPI use to uncommon but serious adverse health outcomes, including hip fracture, community acquired pneumonia, *Clostridium difficile* infection, acute interstitial nephritis and AKI’. More recently they have also been associated with dementia [21, 22]. While each is individually plausible, such a broad spectrum of associated adverse outcomes all with similar and small effect sizes raises a concern that the findings could relate to the same sources of bias.

The challenges of appropriately adjusting for confounding by indication in the context of who is prescribed PPIs became apparent during the extensive research into the clinical importance of a pharmacological interaction between clopidogrel and PPIs on vascular outcomes [23]. The majority of observational studies compared people taking PPIs and clopidogrel with those taking clopidogrel alone and found positive associations of the combination therapy with vascular events. However, important differences were observed between people prescribed a PPI and those not prescribed a PPI in terms of risk factors for vascular events [24]. One study compared the results of two study designs derived from the same dataset of patients in UK primary care to investigate the role of residual confounding [25]. The traditional cohort design showed an increased risk of vascular events in people taking PPIs and clopidogrel compared with clopidogrel alone, but tellingly, the cohort study also identified an association between PPIs and harmful outcomes that would not be predicted by the drug interaction (despite adjusting for relevant recorded confounders), pointing towards residual confounding as a possible explanation. In the same study, a self-controlled case series analysis, a method that compares time periods within individuals to remove between-person confounding, showed no increase in risk within individuals when they were taking both drugs. This suggested that the possible observed interaction between clopidogrel and PPIs was not of clinical importance and that findings of harm in the multiple previous cohort studies were explained by confounding by poorer health among people taking PPIs that was difficult to adjust for in standard analyses.

The importance of assessing bias cannot be overstated, and only very thorough investigation with a range of study designs or alternative outcomes will unveil its influence. Alternative negative outcomes to understand the degree of residual confounding in future studies of the PPI–CKD question may be useful, for example, incident depression or cataract surgery. Because CKD is not an acute event, a self-controlled case series design is not suited to the study of CKD. A self-controlled case series of the association of PPI use with AKI has not yet been conducted but may be informative.

## CONCLUSION

Bearing in mind the history of PPI-associated adverse event research and the potential sources of bias, we do not believe that these papers on their own provide strong evidence of a causal link with CKD. This caution has also been expressed by the authors, e.g. Lazarus *et al.* [1] state ‘further research is required to investigate whether PPI use itself causes kidney damage’, while Xie *et al.* [2] state ‘the findings should not deter from prescription and use of PPI where medically indicated’. Nonetheless, there is no doubt that many patients remain on PPIs for long periods with no clear indication. Studies such as these serve as salutary reminders to constantly review whether all drugs remain indicated for each patient, and to de-prescribe if necessary. Stopping PPIs can cause an exacerbation of severe dyspeptic symptoms, and inevitably some patients may suffer more severe complications, for instance, upper GI bleeding (as one of the high-risk consequences), after cessation.

One may argue that decisions about prescribing are best informed by an overall risk–benefit analysis derived from well-conducted clinical trials. However, in reality, many such trials will not be feasible (e.g. for rare harms), nor affordable, nor always ethical. If done well, observational data have a great deal to contribute to our understanding of the incidence and causes of prescribing-related adverse outcomes in routine clinical care.

## CONFLICT OF INTEREST STATEMENT

All authors report no conflict of interest. None of the authors have directly worked for companies that produce or market PPIs. D.G.F. has done event adjudication via a research management company for the CARMELINA Trial but at no point directly communicated with Novartis. The results presented in this paper have not been published previously in whole or part.
